# The snoRNA MBII-52 Regulates Cocaine-Induced Conditioned Place Preference and Locomotion in Mice

**DOI:** 10.1371/journal.pone.0099986

**Published:** 2014-06-30

**Authors:** Hongjie Chen, Hao Qiang, Kaichun Fan, Shousen Wang, Zhaocong Zheng

**Affiliations:** 1 Department of Neurosurgery, Fuzhou General Hospital, Second Military Medical University, Fuzhou, Fujian, China; 2 Department of Radiology, Changhai Hospital, Second Military Medical University, Shanghai, China; 3 Department of Gastroenterology, Chinese PLA General Hospital, Beijing, China; Universidade de São Paulo, Brazil

## Abstract

Cocaine dependence involves in the brain's reward circuit as well as nucleus accumbens (NAc), a key region of the mesolimbic dopamine pathway. Many studies have documented altered expression of genes and identified transcription factor networks and epigenetic processes that are fundamental to cocaine addiction. However, all these investigations have focused on mRNA and/or miRNA, which may not reflect the involvement of small nucleolar RNAs (snoRNAs), which has been implied in a broad range of biological processes and complex diseases including brain development and neuropathologocal process. To further address the role of snoRNA in cocaine addiction, we show that repeated exposure and conditioned place preference (CPP) training to cocaine negatively regulates the expression of MBII-52 mRNA level, which is a brain-specific C/D box snoRNA, but not influences the serotonin receptor 2C (5HT2CR) mRNA level in NAc. Furthemore, we show, developing lentiviral vector (LV)-expressing MBII-52 and LV-5HT2CR for stable and regulatable MBII-52 and LV-5HT2CR expression. LV-MBII-52 and LV-5HT2CR expression in NAc attenuate cocaine induced CPP and locomotor activity. Taken together, these findings show that MBII-52 and 5HT2CR exert an inhibitory influence on the behavioral responses to cocaine exposure.

## Introduction

Cocaine addiction is defined as a psychological disease that is characterized by uncontrollable, compulsive drug seeking and drug abuse, which poses negative health and social consequences [1], [2]. Repeated cocaine exposure produces changes in gene expression and altered neuronal morphology within the nucleus accumbens (NAc), a brain region involved in the motivational effects of drugs abuse [3], [4], and these alterations are thought to contribute to the expression of conditioned place preference (CPP) and behavioral sensitization, which have been widely used for underlying the rewarding effect of psychostimulants [5], [6].

Small nucleolar RNAs (snoRNAs) represent one of the most abundant noncoding RNA (ncRNA) molecules of ∼60 to 300 nucleotides in length [7]. Two main groups of snoRNAs have been described, C/D box and H/ACA box snoRNAs [8], both snoRNA classes exert microRNA-like silencing function by base-pairing to complementary sites within target rRNAs or small nuclear RNAs (snRNAs) [7]. In previous studies, high-throughput screening for ncRNAs in mice revealed that MBII-52 is a brain-specific C/D box snoRNA [8], [9]. Interestingly, MBII-52 has been reported to exhibit an 18-nucleotide-long complementarity to an alternative exon of the serotonin receptor 2C (5HT2CR) pre-mRNA [9]. In the past, several groups have reported the MBII-52 involved in regulation of editing or splicing of the 5HT2CR pre-mRNA in the Prader-Willi syndrome (PWS) [10], [11]. In these studies, it has been noted that a mouse model lacking MBII-52 snoRNAs shows differences in pre-mRNA processing of the serotonin receptor. Furthermore, 5HT2CR has been identified as a key regulatory receptor in dopamine neurotransmission and cocaine pharmacology [12], [13]. Administration of 5HT2CR agonists, RO 60-0175, attenuated cocaine-induced CPP and locomotor sensitization [14]. Based on the accumulating evidence, a hypothesis is that dysregulation of snoRNA in the brain could contribute to fundamental aspects of cocaine addiction. Moreover, the role of MBII-52 in the cocaine-induced behavioral plasticity has not been addressed so far.

In the present study, we found that repeated exposure and CPP to cocaine negatively regulates the expression of MBII-52 mRNA level, but not influences 5HT2CR mRNA level in NAc. Increased MBII-52 or 5HT2CR expression in NAc attenuates the development of cocaine-induced CPP and locomotor sensitization. Thus, results from the present study show that repeated cocaine exposure induced downregulation of MBII-52, which correlates cocaine behavioral rewarding and sensitized.

## Materials and Methods

### 1. Animals

Adult 8- to 10-week-old male C57BL/6J mice housed 5 per cage were maintained in a temperature-controlled vivarium (21±2°C) on a 12 h light-dark cycle. Food and fluid were available ad libitum. All animal protocols in this study were in accordance with the guidelines established by the Association for Assessment and Accreditation of Laboratory Animal Care. The protocols were approved by the committee on the ethics of animal experiments of Fuzhou General Hospital of Second Military Medical University (Protocol number IACUC-S201104-P03).

### 2. Drugs

Cocaine hydrochloride (cocaine-HCl) was provided from the National Institute for the Control of Pharmaceutical and Biological Products (Beijing, China). Cocaine was dissolved in 0.9% saline and administered intraperitoneally (i.p.) in a volume of 10 ml/kg body weight.

### 3. Cocaine treatments

Adult male 8- to 10-week-old C57BL/6 mice received seven daily injections of saline, acute cocaine treatments (six saline injections plus one 20 mg/kg cocaine-HCl injection) or repeated cocaine treatments (seven 20 mg/kg cocaine-HCl injections). The NAc of mice were used for detecting expression of MBII-52 or 5HT2CR mRNA at 24 h after the last injection.

### 4. Conditioned place preference procedure

CPP test in mice was similar to that previously described [15], [16]. Briefly, mice were conditioned using a standard CPP paradigm. The standard protocol involved three parts: pretest, conditioning and test. The animals were acclimatized for 7 days before experiment and habituated to handling for three days before behavioral testing in order to adapt to environment. During pre-conditioning phase (day 1, pretest), mice were placed in the central area and allowed to explore both compartments freely for 15 min. The time spent in each chamber was recorded, and the mice were randomly arranged into control group and cocaine group with equivalent pretest scores. After injection of cocaine in cocaine group (20 mg/kg, i.p.) and injection of saline in control group (0.9% sodium chloride) on days 2, 4 and 6, mice were confined to the corresponding conditioning compartment by closing the removable wall for a period of 15 min. After injection of saline in both cocaine group and saline group on days 3, 5 and 7, all mice were confined to the opposite conditioning chamber for the same time. In post-conditioning phase (day 8, test), mice were placed as in the central area with free access to both compartments for 15 min and the time spent in each compartment was measured. Results were expressed as the time (in seconds) spent in the cocaine-paired chamber minus the time spent in the saline-paired chamber during CPP testing.

### 5. Locomotor activity

Locomotor activity sessions were conducted once daily. Each mouse was placed in a locomotor activity chamber followed by cocaine (20 mg/kg, i.p.) or saline and the locomotor activity was measured for 30 minutes as previously described with slightly modifications [17]. The chambers were black acrylic boxes (40.64 cm×40.64 cm×31 cm) that were equipped with a top unit including a camera. Automated tracking were performed using EthoVision 7.0 software (EthoVision 7.0; Noldus Information Technology, Leesburg, VA).

### 6. RNA isolation and quantitative PCR

NAc punch dissections were homogenized in Trizol (Invitrogen) and processed according to the manufacturer's instructions. RNA was purified with RNAesy Micro columns and spectroscopy confirmed that the RNA 260/280 ration was >1.8. RNA was then reverse transcribed using a Bio-Rad iScript Kit. The cDNA was quantified by qPCR using SYBR Green. Each reaction was run in triplicate and analyzed following the ΔΔCt method. Data were analyzed as for qPCR using 18S rRNA as the housekeeping gene (GAPDH and ACTIN were also tested) [11]. The following primer pairs were used:

MBII-52 F: GGGTCAATGATGACAACCCAATG

MBII-52 R: GGGCCTCAGCGTAATC CTA TTG

5HT2CR F: CATCATGAAGATTGCCATCGTT

5HT2CR R: CGCAGGTAGTATTATTCACGAACACT

18s rRNA F: GTAACCCGTTGAACCCCATT

18s rRNA R: CCATCCAATCGGTAGTAGCG

### 7. Construction of lentiviral vectors for overexpression of MBII-52

pLV-CMV>MBII-52-EGFP and pLV-CMV-EGFP virus plasmids were constructed for the production of lentiviruses expressing MBII-52 and GFP (LV-MBII-52) and lentivirus expressing GFP (LV-GFP). These two vectors contained the enhanced green fluorescence protein (GFP) reporter under the control of a cytomegalovirus (CMV) promoter. All vector insertions were confirmed by dideoxy-sequencing. Recombinant lentiviruses were produced by transient transfection in HEK293T cells, using pLV-CMV>MBII-52-EGFP (to yield LV-MBII-52) and pLV-CMV-EGFP (to yield LV-GFP). Viral titers were determined by infection of 293T cells and GFP visualization (1.02×10^8^ TU/ml). Aliquots were kept at −80°C. LV-5HT2CR viruses were similarly prepared. Image was obtained using standard flurescent microscopy.

### 8. Stereotaxic surgery

Under general ketamine/xylazine anesthesia, mice were positioned in a small-animal stereotaxic instrument, and the cranial surface was exposed. Thirty-three gauge syringe needles were used to bilaterally infuse 0.5 µl of virus into the NAc at a 10° angle (anterior/posterior +1.6 mm; medial/lateral +1.5 mm; dorsal/ventral −4.4 mm from bregma) at a rate of 0.1 µl/min. Animals receiving LV injections were allowed to recover for one week after surgery before beginning the CPP or locomotor activity to cocaine as described above.

### 9. Statistical analysis

Data were analyzed by two-way analysis of variance (ANOVA) with repeated measures for CPP and repeated-measures ANOVAs were used for locomotor activity, and one-way ANOVA following by Tukey post hoc test was used to determine significance for RT-PCR experiments with greater than two groups. All values included in the figure legends represent mean ± SEM. The significance level was set at P<0.05.

## Results

### 1. Transcriptional regulation of MBII-52 and 5HT2CR in NAc by cocaine

**Figure 1:**
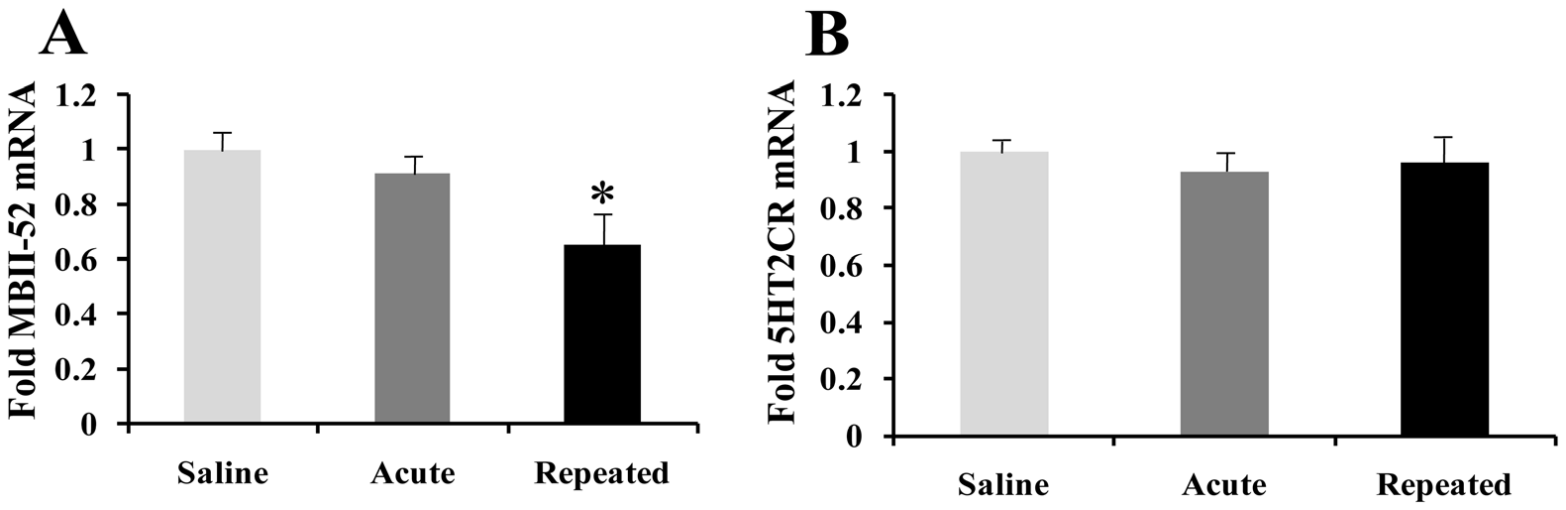
Transcriptional regulation of MBII-52 and 5HT2CR by chronic cocaine in NAc. Repeated cocaine administration, but not acute, significantly decreased MBII-52 (A) and not 5HT2CR (B) mRNA in NAc 24 h after the final drug injection compared with saline treatments (n = 6 mice per group; *p<0.05).

We performed quantitative PCR (qPCR) on NAc tissue of mice treated acutely or chronically (seven daily injections) with cocaine for MBII-52 and 5HT2CR (Fig. 1). As a first step, we investigated whether cocaine regulates the expression of MBII-52 in the mice NAc in *vivo*. Repeated cocaine administration, but not acute significantly induced downregulation of the MBII-52 expression in NAc at 24 h (cocaine: F[2,18] = 3.21; P<0.05) after the last cocaine infusion (Fig. 1A). To identify a mechanism by which cocaine downregulates MBII-52 expression, we examined the 5HT2CR mRNA expression. The 5HT2CR expression is unchanged 24 h after repeated or acute cocaine administration (Fig. 1B) in NAc. Although little is known about the potential of MBII-52 in cocaine induced behavioral adaption, its unique function in editing of exon Vb changes the amino acid sequence of the 5HT2CR [10] not the mRNA expression, led us to presume that MBII-52 repression elicited by cocaine in NAc could be involved in cocaine reward and neuroplasticity.

### 2. Effect of cocaine CPP training and testing on MBII-52 and 5HT2CR mRNA levels in NAc

**Figure 2:**
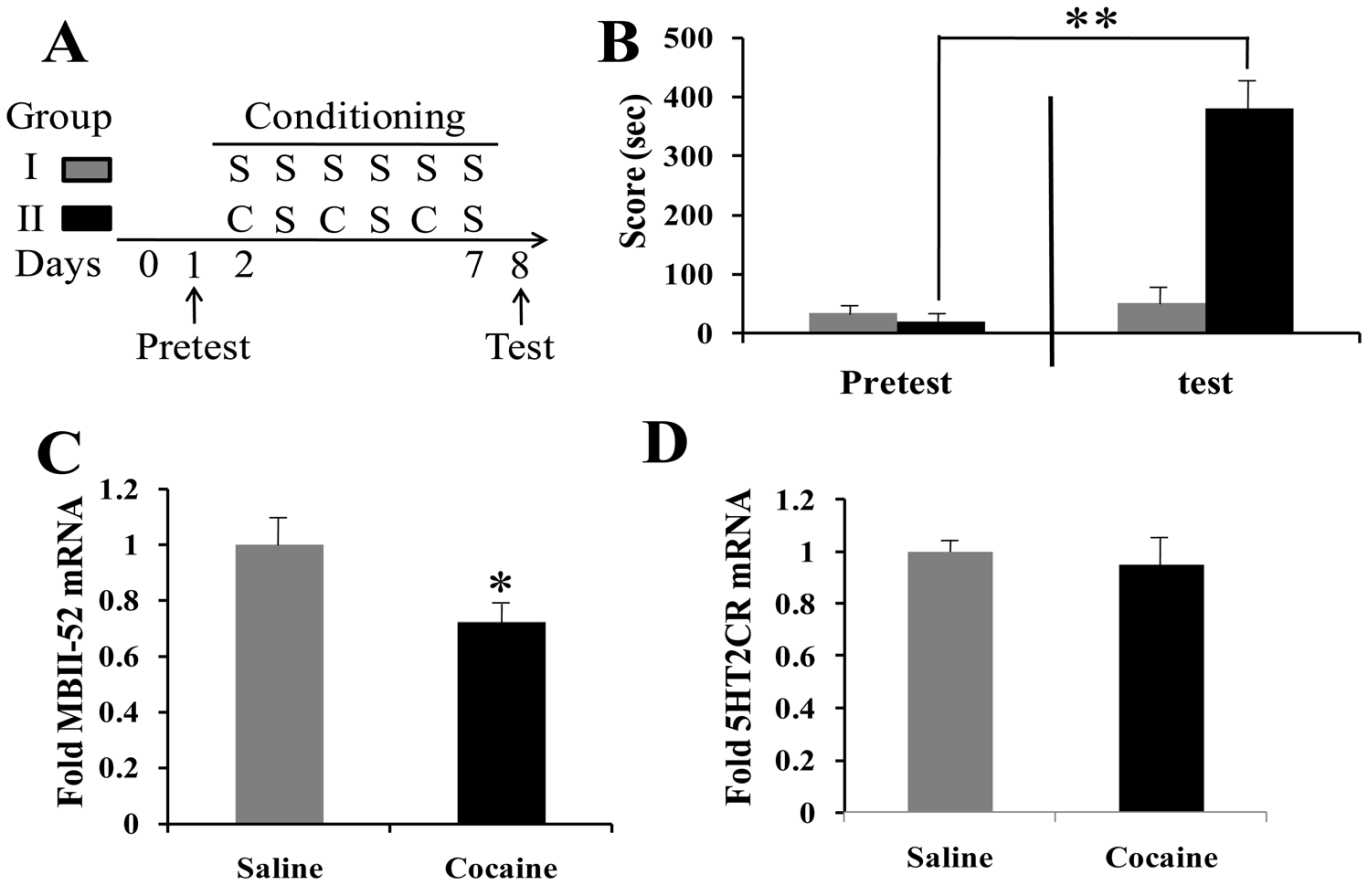
Cocaine CPP represses MBII-52 and 5HT2CR expression in NAc. (A) Timeline of cocaine CPP experiment. Experimental design: S, saline; C, cocaine. (B) Conditioned place preference for cocaine. After conditioning, mice developed a significant preference for the cocaine-paired side. (n = 8, **P<0.01). Decreased expression of MBII-52 (C) and not 5HT2CR (D) mRNA in the NAc after cocaine-induce CPP. n = 8 and *P<0.05.

To examine the expression of MBII-52 and 5HT2CR mRNA levels in NAc, mice were conditioned to cocaine in a 6-day unbiased CPP protocol (Fig. 2A, group II) and compared with mice receiving saline injections (group I). The rate of time spent in drug-paired compartment during the place preference test was presented (Fig. 2B). Each group spent about 50% time on the drug-paired side during the pre-conditioning phase, confirming the unbiased nature of our place conditioning procedure. The calculated CPP score for the saline group was 52±14 seconds and the cocaine-paired groups was 382±49 seconds (Fig. 2B). The group received 20 mg/kg of cocaine spent significantly more time in the cocaine-paired chamber on the test day (Day 8) than the pretest day (Day 1) (P<0.01). The mice were decapitated and their brains were removed for qPCR analysis after the test session of CPP test immediately. We found that CPP training decreased MBII-52 mRNA levels in NAc (cocaine: F[5,16] = 2.76; P<0.05), and no significant effects were observed for 5HT2CR levels in NAc (cocaine: F[2,16] = 0.58; P>0.05) (Fig. 2C, D).

### 3. MBII-52 in the NAc regulates cocaine reward behavior

**Figure 3:**
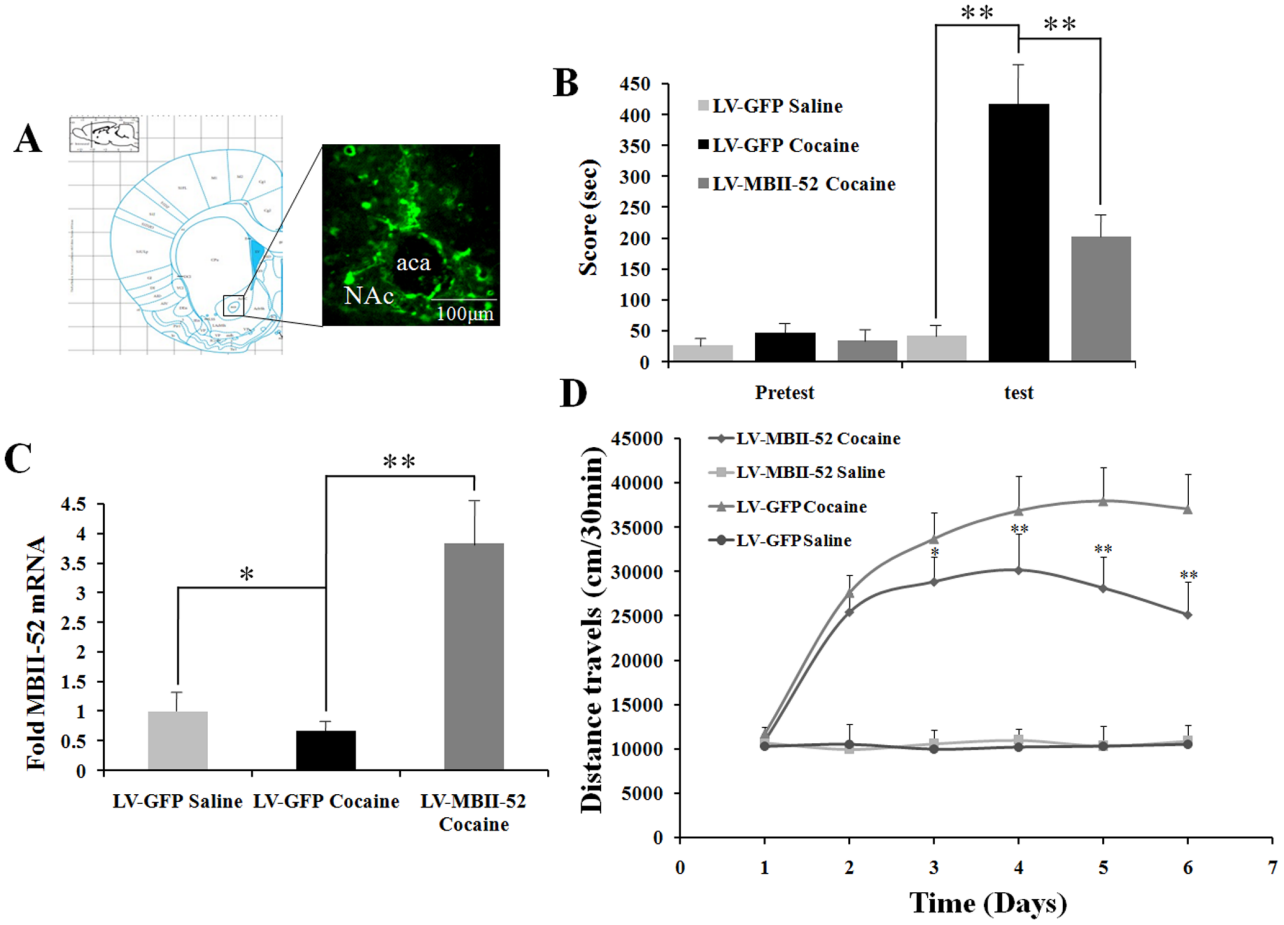
Virally expressed MBII-52 in the NAc regulates cocaine-induced CPP and locomotor activity. (A) Verification of anatomical placement and viral infection in NAc after LV-MBII-52 EGFP injection; immunostaining for GFP is shown. (B) Virally expressed MBII-52 significantly attenuated cocaine-induced CPP. n = 10, *P<0.05 and **P<0.01. (C) MBII-52 mRNA level in the NAc of mice infected with LV-MBII-52 after cocaine CPP treatment. *P<0.05 and **P<0.01. (D) LV-MBII-52 significantly reduced the locomotor activity of the mice with repeated cocaine administration. n = 10, *P<0.05 and **P<0.01.

To understand the behavioral importance of MBII-52 downregulation in NAc after repeated cocaine exposure or cocaine-induced CPP, mice were received intra-NAc injections of lentiviruses expressing MBII-52 (LV-MBII-52) to temporally and specifically overexpress MBII-52 in the NAc (Fig. 3A), and then evaluated whether such genetic manipulation could block cocaine-induced CPP, which provides an indirect measure of cocaine reward [18], [19]. Importantly, MBII-52 overexpression in this brain region markedly attenuated the preference for cocaine in comparison to that seen in animals expressing GFP (Fig. 3B). It revealed a difference between the group conditioned with cocaine (LV-GFP) and treated with saline (LV-GFP) (Cocaine: F[1,27] = 28.81; P<0.01) and the group conditioned with cocaine (LV-GFP) and the other group conditioned with cocaine (LV-MBII-52) (LV-MBII-52 infusion: F[1,27] = 11.37; P<0.01; Cocain×LV-MBII-52 infusion: F[3,27] = 3.41; P<0.05). To investigate whether such behavioral change correlated with alterations of MBII-52 expression, we detected the levels of MBII-52 mRNA in NAc after CPP regimen. We found that the MBII-52 mRNA levels significantly elevated (Fig. 3C). No significant group differences were observed during the saline exposure to the locomotor activity chambers between the LV-GFP group and LV-MBII-52 group. Moreover, LV-MBII-52 overexpression did significantly impair locomotor sensitization to repeated cocaine administration (Day: F[3,37] = 14.31, P<0.01; LV-MBII-52 infusion: F[1,27] = 27.34; P<0.01; Day×LV-MBII-52 infusion: F[3,27] = 4.85; P<0.05), with significantly lower locomotor activities observed on days 4, 5, and 6 of cocaine treatments (P<0.01) (Fig. 3D).

### 4. 5HT2CR overexpression in NAc also regulates cocaine reward behavior

**Figure 4:**
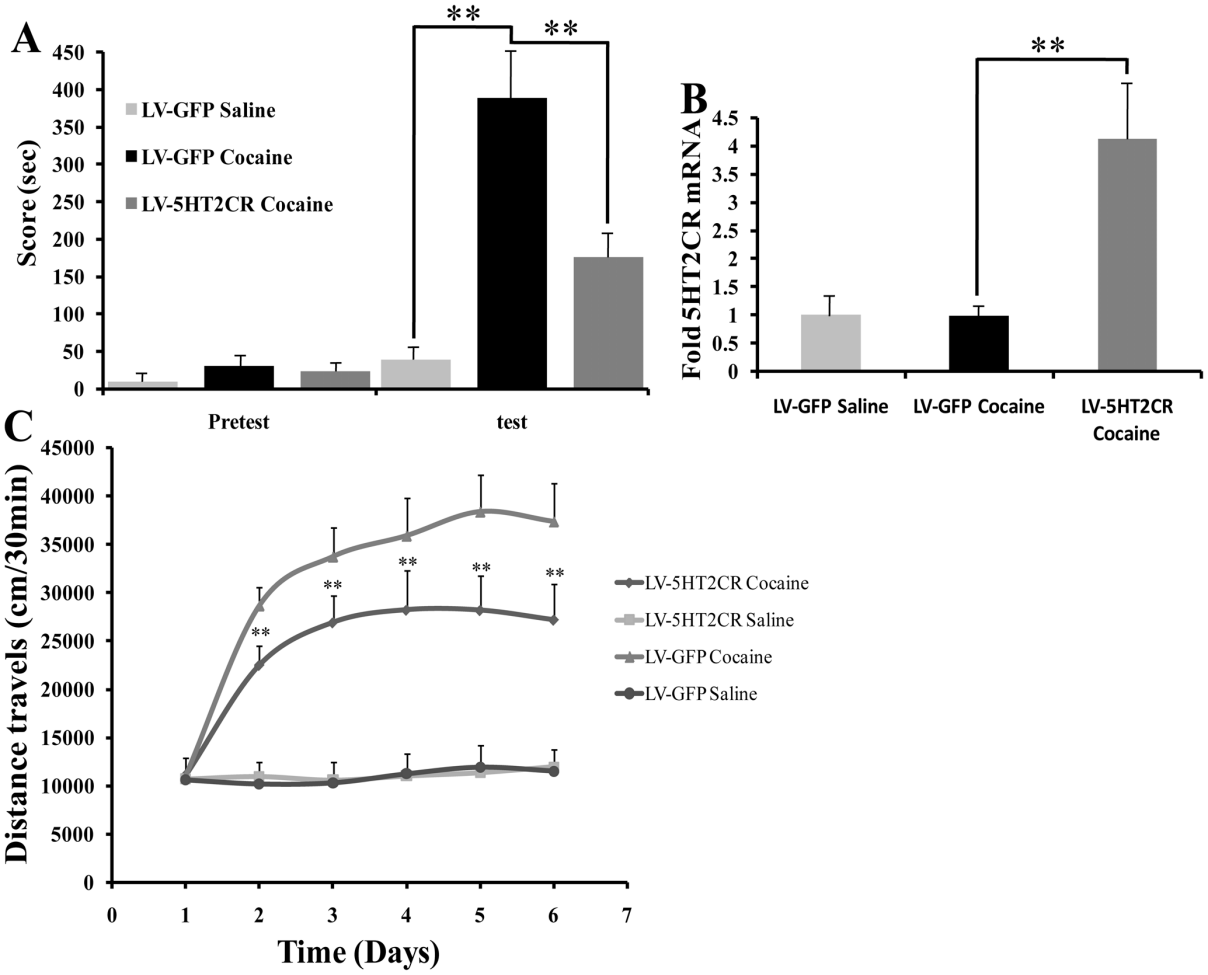
Virally expressed 5HT2CR in the NAc regulates cocaine-induced CPP and locomotor activity. (A) Virally expressed 5HT2CR significantly attenuated cocaine-induced CPP. n = 10, **P<0.01. (B) 5HT2CR mRNA level in the NAc of mice infected with LV-5HT2CR after cocaine CPP treatment. **P<0.01. (C) LV-5HT2CR significantly reduced the locomotor activity of the mice with repeated cocaine administration. n = 10, **P<0.01.

To further study the role of MBII-52 in the behavioral effects of cocaine, the mice received intra-NAc injections of lentiviruses expressing 5HT2CR recombinase or GFP as a control, verification of viral infection expressing 5HT2CR was similar to Fig. 3A (data not show). LV-5HT2CR overexpression in NAc significantly decreased the effects of cocaine-induced CPP and locomotor activity (Fig. 4 A, C). It revealed a difference between the group conditioned with cocaine (LV-GFP) and treated with saline (LV-GFP) (Cocaine: F[1,27] = 25.35; P<0.01) and the group conditioned with cocaine (LV-GFP) and the other group conditioned with cocaine (LV-5HT2CR) (LV-5HT2CR infusion: F[1,27] = 8.61; P<0.01; Cocaine×LV-5HT2CR infusion: F[3,27] = 2.12; P<0.05). Significant main effects for locomotor activity were revealed (Day: F[3,37] = 9.72, P<0.01; LV-5HT2CR infusion: F[1,27] = 16.64; P<0.01; Day×LV-5HT2CR infusion: F[3,27] = 2.95; P<0.05). No statistical difference between the cocaine group and saline group expressing GFP, and there were significant lower locomotor activities between the group with LV-5HT2CR or without on Day 2 to Day 6 of cocaine treatments (P<0.01) (Fig. 4B). These behavioral data suggest that increased MBII-52 or 5HT2CR mRNA expression in NAc negatively regulated cocaine-induced CPP and locomotor activity, whereas decreased MBII-52 enhances cocaine reward and locomotor activity.

## Discussion

The ncRNA, especially miRNA, have been implicated in chronic psychiatric conditions and as a potential mechanism by which psychostimulants causes long-lasting changes in gene expression within mature neurons and behaviors [20], [21]. However, there are no studies concerning the role of snoRNA in neuroadaptations. Here we show that MBII-52, a brain-specific C/D box snoRNA, is downregulated by repeated cocaine exposure and cocaine-induced CPP. Our functional experiments indicate that MBII-52 and 5HT2CR play critical roles in cocaine behavioral plasticity. Our study, for the first time, provides a new insight on current model that snoRNA in the NAc has a pronounced effect on cocaine-induced behavior plasticity.

NcRNAs have been shown to be a critical factor in mechanisms underlying cellular and behavioral responses to cocaine [21], [22]. The first groundbreaking study demonstrated that alterations in miRNA directly affected cocaine-dependent expression of specific genes, as well as the behavioral responses [21]. For example, cocaine self-administration in rats reportedly increases expression of the miR-212 in striatum, and experimentally increasing miR-212 levels in this region decreases cocaine reward [21]. These results suggest that ncRNAs involved in the formation of context-cocaine associated memories similar to observations showing that ncRNAs regulated immediately after training enhances cocaine reward [23]. These results indicate that changes in ncRNAs are required for cocaine-induced behavior plasticity. Tthe CMV promoter was stronger than other promoters in the brain area using LV vectors [24], [25] and the CMV promoter is widely used in the previous study in cocaine exposure [19], [21], [26], so the CMV promoter was used to overexpress the MBII-52 or 5HT2CR in this study. CPP is often used to measure the reinforcing properties of psychotropic drugs such as cocaine [27]. It involves the association between environmental and reinforcing stimuli produced by the drug. Thus, it demonstrated great utility in the search for new mechanisms for drug abuse and dependence. Regarding our study, cocaine at a dose of 20 mg/kg successfully produced a positive CPP. Additionally, the overexpression of MBII-52 or 5HT2CR significantly reduced the CPP scores and locomotor activity.

Due to an 18-nucleotide sequence complementarity, HBII-52 can bind to the alternatively spliced exon Vb of the 5HT2CR pre-mRNA, where it masks a splicing silencer, which results in alternative exon usage [10]. This silencer can also be destroyed by RNA editing, which changes the amino acid sequence and appears to be independent of HBII-52. Lack of HBII-52 expression in individuals with PWS causes most likely a lack of the high-efficacy serotonin receptor, which could contribute to the disease. Recently, evidence from using the PWS-IC^+/−^ mouse model, appear to MBII-52 regulate brain serotonin effects on behavior via its action on 5HT2CR [11]. The 5HT2CR is a key regulatory receptor of serotonin and dopamine neurotransmission has been found in previous studies [28], [29]. According to use microdialysis techology in vivo, they found the agonist and antogonist of 5HT2C regulate cocaine-induced alterations in dopamine neurotransmission in different brain area, such as the NAc and ventral tegmental area [13]. Moreover, some studies have shown that administration of 5HT2CR agonists prior to cocaine regulates cocaine-induced CPP and sensitization [14] and self-administration [30]. The results presented here are partially in accordance with our hypothesis that MBII-52 could act on the editing of the 5HT2CR to attenuate cocaine-induced CPP and locomotor activity.

In summary, we report for the first time a role for the MBII-52, which correlates with both the rewarding and locomotor stimulating effects produced by cocaine. The MBII-52 exhibits an inhibitory mode of action over behavioral responses to cocaine, potentially through 5HT2CR mechanism. Future studies will further address the link between MBII-52 and 5HT2CR, and the underlying neurocircuitry occurring via MBII-52 and 5HT2CR interactions in the context of cocaine exposure.
